# Supplementary material for: Genetic and pharmacological inhibition of microRNA-92a maintains podocyte cell cycle quiescence and limits crescentic glomerulonephritis
Source: Nat Commun. 2017 Nov 28;8:1829. doi: 10.1038/s41467-017-01885-7 (PMC5705755; doi:10.1038/s41467-017-01885-7)
Supplement: Supplementary file 1 — Supplementary Information [file 41467_2017_1885_MOESM1_ESM.pdf]

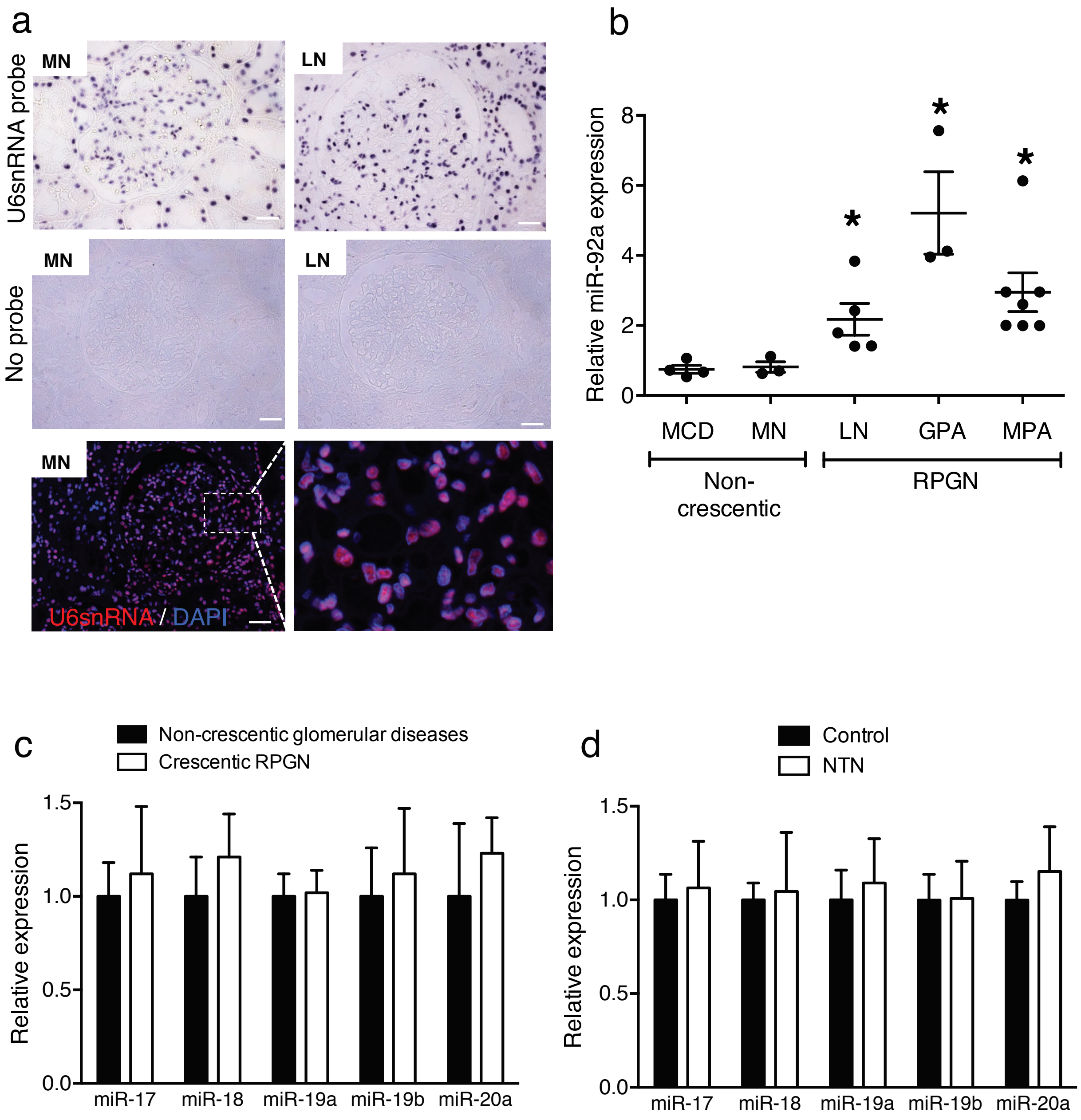

Supplementary Figure 1: ***High expression of miR-92a in human kidneys with RPGN***

(**a**) Representative photomicrographs of visible or fluorescent U6snRNA, as a positive control, *in situ* hybridization of kidney sections from patients. (**b**) RT-qPCR analysis of the relative abundance of miR-92a in biopsies from 7 studied patients with noncrescentic glomerulopathies (left) and 15 patients with RPGN of various etiologies including lupus nephritis (LN), microscopic polyangiitis (MPA) and granulomatosis with polyangiitis (GPA). (**c**) RT-qPCR analysis of the relative abundance of miR-17, miR-18a, miR-19a, miR-19b and miR-20a in biopsies of studied patients. (**d**) RT-qPCR analysis of the relative abundance of miR-17, miR-18a, miR-19a, miR-19b and miR-20a on isolated podocytes from NTS-challenged mice (NTN) or controls.

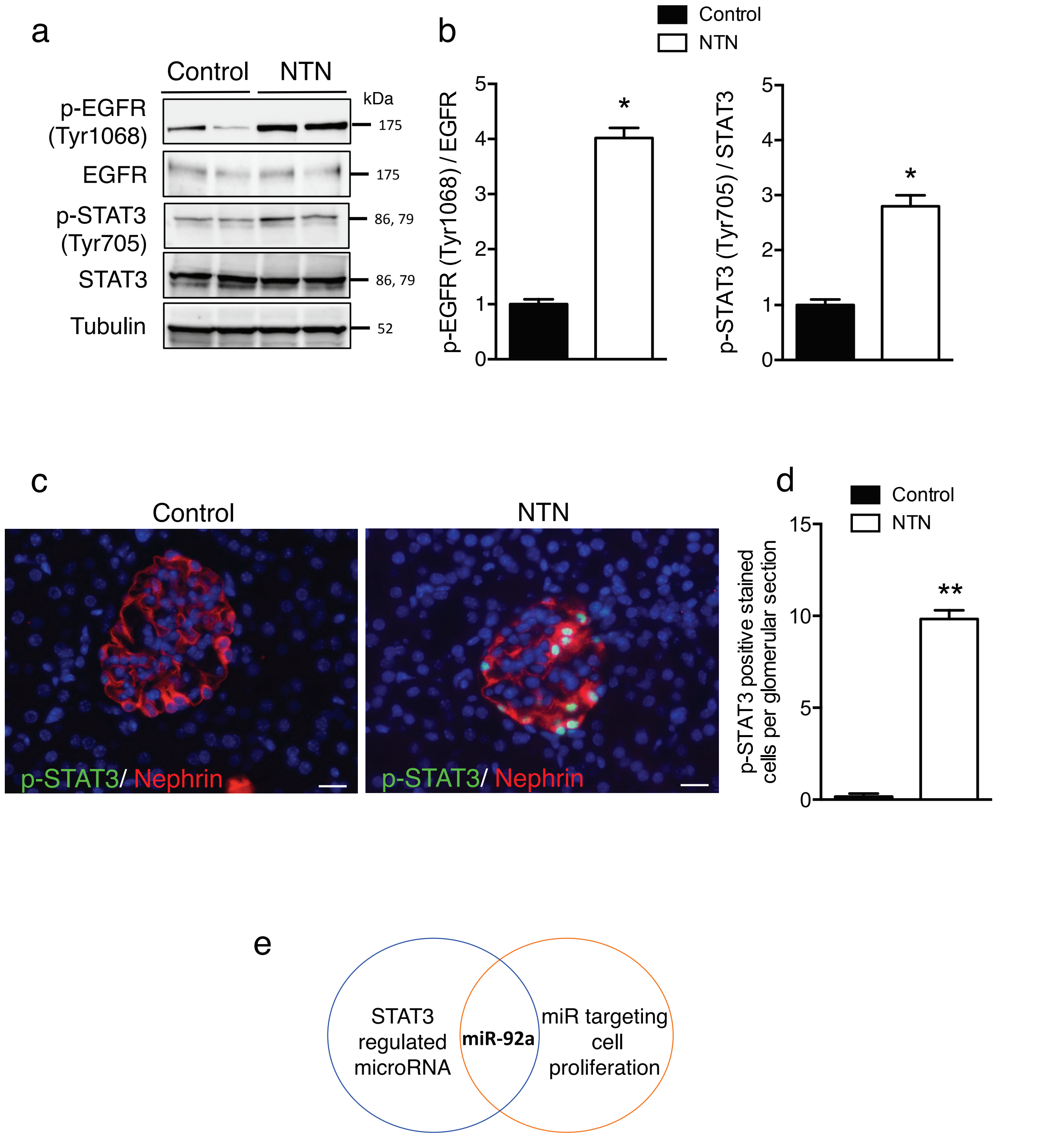

Supplementary Figure 2: ***EGFR and STAT3 activation in the glomerulus during NTN in mice***

(**a,b**) Western blotting (**a**) and quantification (**b**) of phospho-EGFR (Tyr1068), total EGFR, phospho-Stat3 (Tyr705) and total STAT3 abundance in dynabeads isolated glomeruli from control or NTS-challenged mice (NTN). Tubulin is shown as a loading control. Values are means ± sem from at least five mice per condition. * $p < 0.05$ versus unchallenged mice (control). (**c,d**) Representative images of immunostaining for phospho-STAT3 (Tyr705) (green) and nephrin (red) (**c**) and quantification in kidney sections of control or NTS-challenged mice (**d**). Scale bars, 10µm. (**e**) Strategy scheme to select candidate microRNA

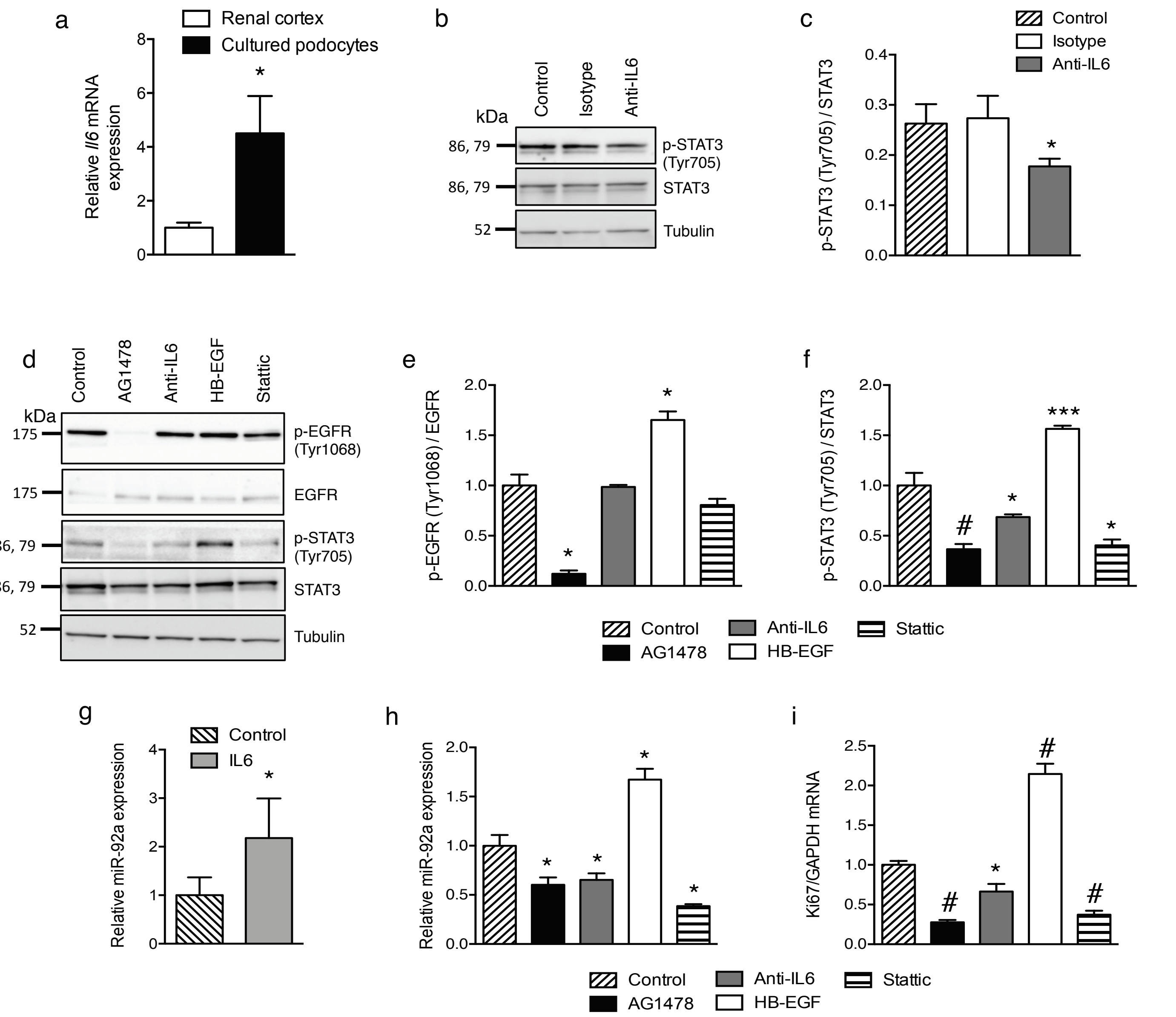

Supplementary Figure 3: ***miR-92a abundance is controlled by EGFR and IL-6 pathways via STAT3 signaling in podocytes***

(**a**) Relative abundance of miR-92a in primary culture of podocytes and normal kidney cortex. Values are means ± sem (n = 3 per condition, 3 independent experiments). * p < 0.05 versus cortex. (**b,c**) Representative Western blotting results (**b**) and quantification of phospho-STAT3 (Tyr705) on total STAT3 signals (**c**) in primary cultures of podocytes 16 hours after the addition of anti-mIL-6 monoclonal antibody or isotype control antibody. Values are means ± sem (n = 4, 2 independent experiments). * p < 0.05 versus untreated podocytes (control). (**d-f**) Western blotting (**d**) and quantification of phospho-EGFR (Tyr1068) on total EGFR (**e**), phospho-STAT3 (Tyr705) on total STAT3 (**f**) in primary cultures of podocytes 16 hours after the addition of AG1478 (EGFR kinase inhibitor), anti-mIL-6 monoclonal antibody, HB-EGF or stattic. Values are means ± sem from at least five western blots. * p < 0.05 versus untreated podocytes (control). Values are means ± sem (n = 4, 2 independent experiments). * p < 0.05, *** p < 0.001, # p<0.05 versus untreated podocytes (control). (**g**) Relative abundance of miR-92a assessed by RT-qPCR in primary mouse podocytes cultures stimulated with recombinant mouse IL6 for 16 hours. (**h**) Relative abundance of miR-92a assessed by RT-qPCR in primary mouse podocytes cultures described in (d). (**i**) RT-PCR analysis of the relative abundance of Ki67 in primary podocyte cultures described in (d). Values are means ± sem (n = 3 per condition, 3 independent experiments). * p < 0.05, # p<0.05 versus untreated podocytes (control).

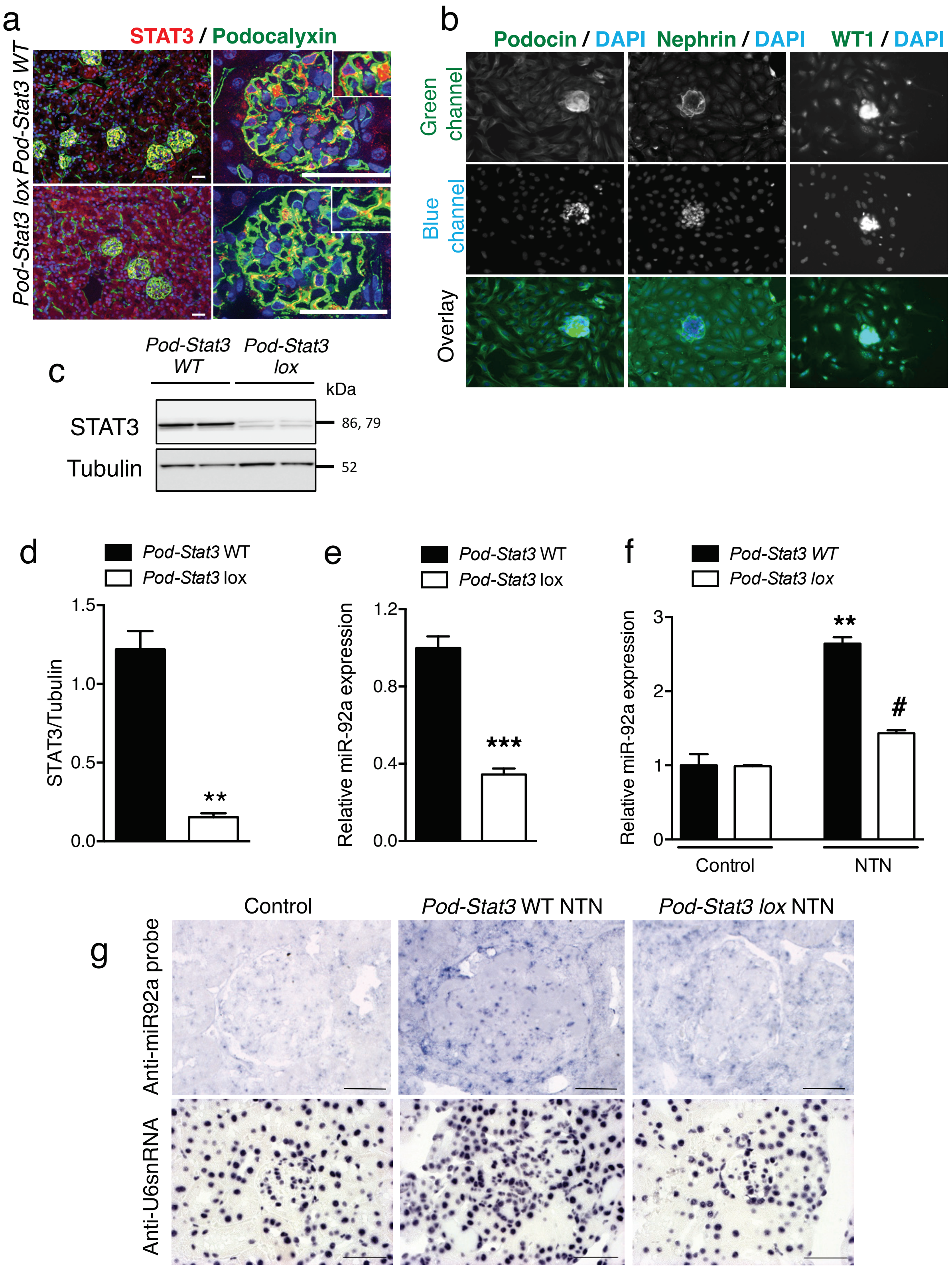

Supplementary Figure 4: *Intact STAT3 signaling cascade in podocytes is required for high miR-92a expression in vitro and in vivo.*

(**a**) Immunofluorescence staining for total STAT3 (red) and podocalyxin (green) in renal cortex from 10-week-old *Pod-Stat3 WT* and *Pod-Stat3 lox* mice. Nuclei are stained with DAPI (blue). Scale bars, 50μm. (**b**) Staining of podocyte markers (podocin, nephrin and WT1, green) in podocyte outgrowths after 4 days of culture. DAPI stained nuclei (blue). Scale bars, 50μm.

Supplementary Figure 4 (continued): ***Intact STAT3 signaling cascade in podocytes is required for high miR-92a expression in vitro and in vivo.***

(**c-d**) Representative western blot image (**c**) and quantification (**d**) of total STAT3 abundance in primary podocyte culture from *Pod-Stat3 WT* and *Pod-Stat3 lox* mice. Tubulin is shown as a loading control. Values are means ± sem (n = 9 mice per group) ** $p < 0.01$ versus *Pod-Stat3 WT* mice. (**e**) Relative abundance of miR-92a assessed by RT-qPCR in primary mouse podocytes cultures from *Pod-Stat3 WT* (control), *Pod-Stat3 lox* mice. *** p < 0.001 versus control podocytes. (**f**) Relative *miR-92a* abundance in isolated glomeruli from unchallenged or NTS-injected *Pod-Stat3 WT* and *Pod-Stat3 lox* mice. Values are means ± sem from at least six mice. ** $p < 0.01$; *** $p < 0.001$ versus non challenged mice and # $p < 0.001$ versus NTS-challenged *Pod-Stat3 WT* mice. (**g**) Representative photomicrographs of miR-92a *in situ* hybridization of kidney sections from unchallenged mice (control), NTS-challenged *Pod-Stat3* WT mice (*Pod-Stat3* WT NTN), NTS-challenged *Pod-Stat3* lox (*Pod-Stat3* lox NTN) after 10 days. U6 snRNA *in situ* hybridization was used as a positive control. Top panel scale bars, 10μm. Bottom panel scale bars 50μm.

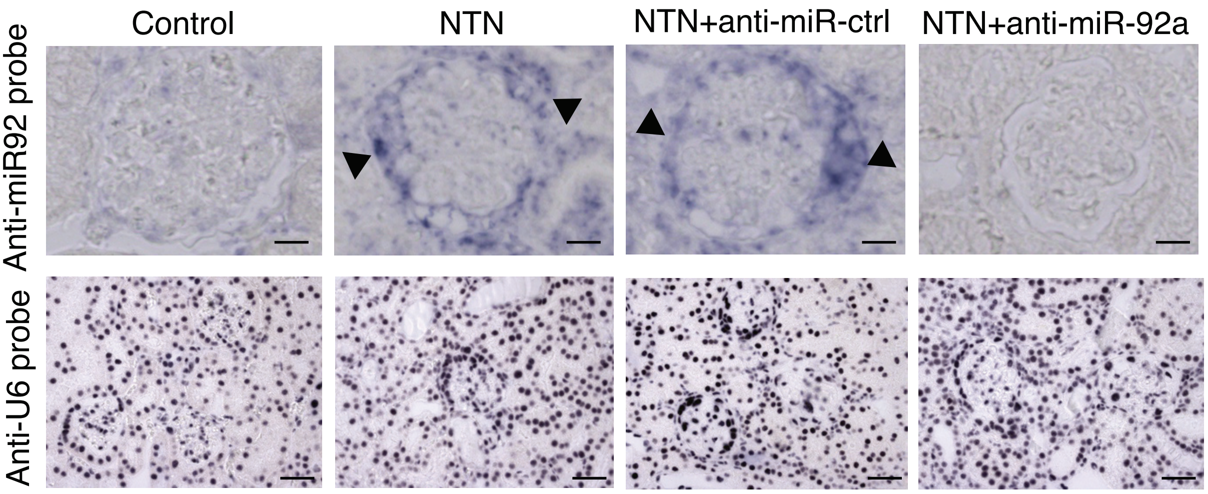

Supplementary Figure 5: *Validation of in vivo miR-92a blockade in mouse model of NTN injected with antagomiR*

Representative photomicrographs of miR-92a *in situ* hybridization of kidney sections from normal mice (control), NTS- challenged mice (NTN), NTS-challenged mice treated with anti-miR-control (NTN + anti-miR-ctrl) and NTS-challenged mice treated with anti-miR-92a (NTN + anti-miR-92a) 10 days after nephrotoxic nephritis induction. Black arrows show strong, specific miR-92a signals in glomerular epithelial cells. Scale bars, 10μm. U6 snRNA *in situ* hybridization is shown as a positive control, scale bars, 20μm.

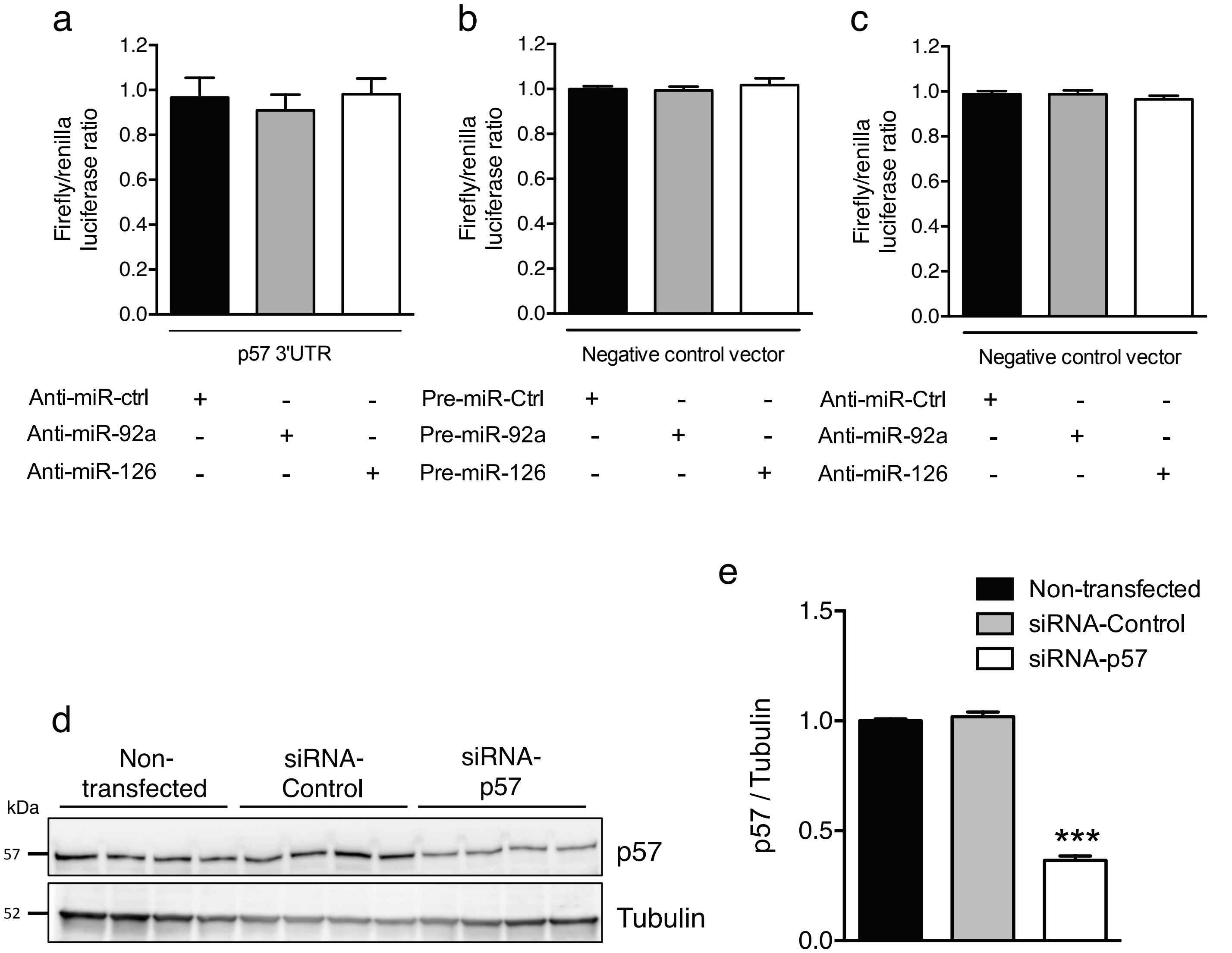

Supplementary Figure 6: ***Validation of p57^{KIP2} as a direct target of miR-92a***

(**a-c**) Dual luciferase assay p57 3'UTR in HEK293 cells transfected with anti-miR-Ctrl, -miR-92a and -miR-126 (**a**), or negative control vector with Pre-miR-Ctrl, miR-92a or miR-126 (**b**) or anti-miR-Ctrl, -miR-92a and -miR-126 (**c**), n=2 independent experiments (each experiment assayed each condition in triplicate). (**d,e**) Western blotting (d) and quantification (e) of p57 on primary podocytes non transfected or transfected with siRNA-p57 or siRNA-control. Tubulin is shown as a loading control. Values are means ± sem from at least three western blots. *** $p < 0.001$ versus untreated podocytes (non transfected).

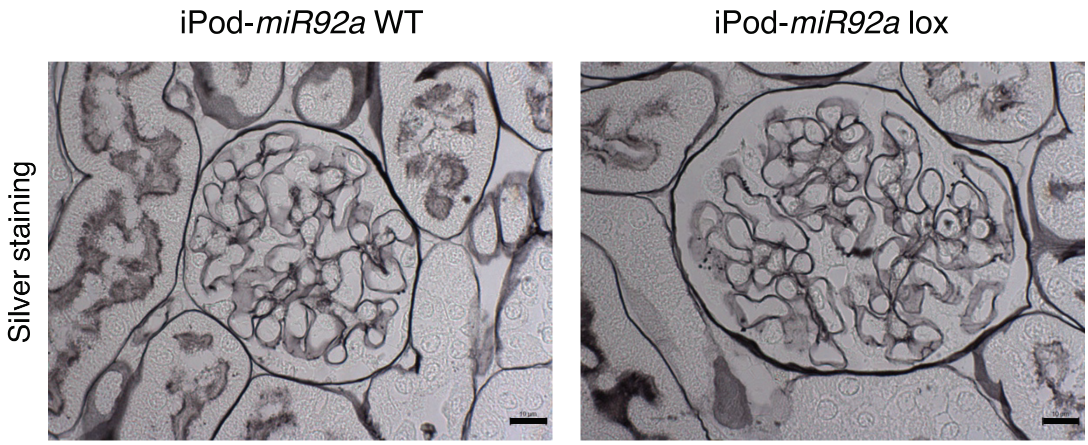

Supplementary Figure 7: ***Normal glomerular morphology in Podocin-rtTA-Tet-O-Cre miR-92a lox mice***

Silver-stained kidney sections of glomeruli from iPod-*miR92a* WT mice and iPod-*miR92a* lox mice at baseline. Scale bars, 10µm.

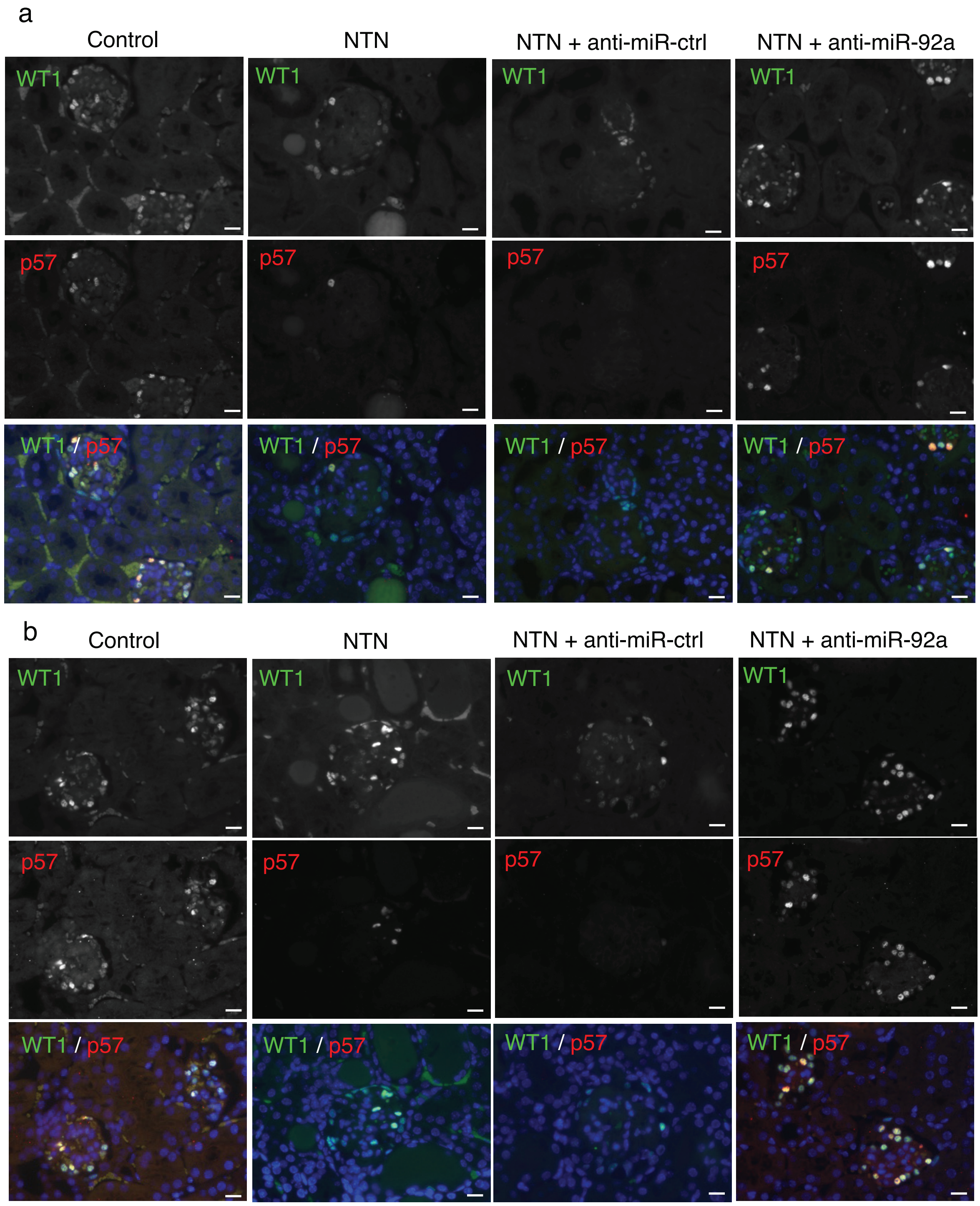

Supplementary Figure 8: ***Altered expression of p57^{KIP2} in podocyte during NTN and antagomiR strategy.***
(**a**) Representative immunofluorescent photomicrographs of WT1 (green) and p57 (red) staining on kidney biopsies from normal mice (control), NTS-challenged mice (NTN), NTS-challenged mice treated with anti-miR-control (NTN + anti-miR-ctrl) and NTS-challenged mice treated with anti-miR-92a (NTN + anti-miR-92a) 10 days after NTN induction. AntagomiR was administrated in a preventative fashion (3 days before NTS injection). (**b**) Representative pictures of immunofluorescent staining of WT1 (green) and p57 (red) on kidney biopsies from normal mice (control), NTS- challenged mice (NTN), NTS-challenged mice treated with anti-miR-control (NTN + anti-miR-ctrl) and NTS-challenged mice treated with anti-miR-92a (NTN + anti-miR-92a) 10 days after nephrotoxic nephritis induction. AntagomiR was administrated with a scheme designed for proof of principle for therapeutic use of anti-miR92a strategies (at day 4 after first NTS injection).

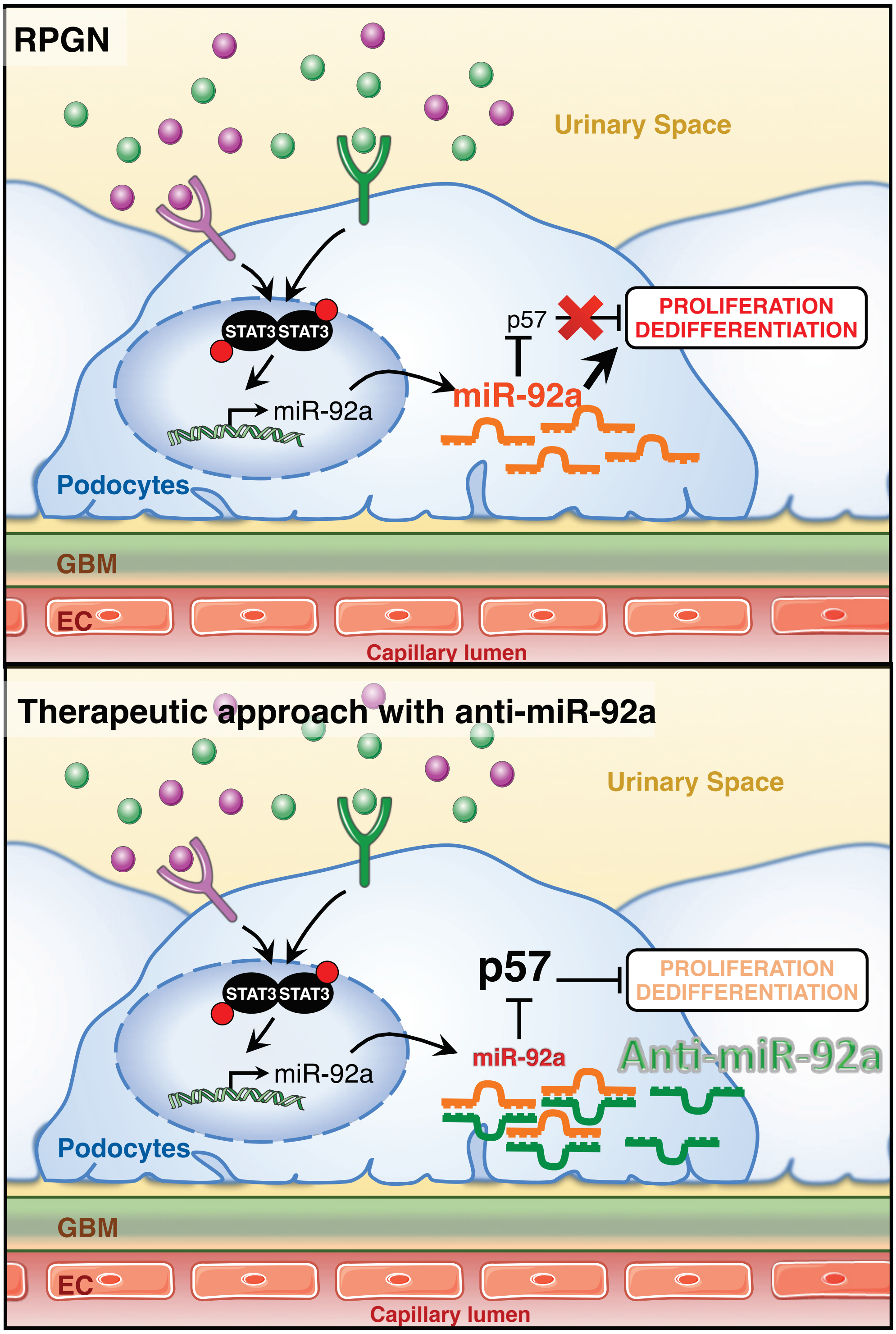

Supplementary Figure 9: ***Working model for the involvement of miR-92a in the pathogeny of crescent formation during RPGN and potential therapeutic strategy***

Various ligands (green dots and purple dots) for podocyte surface receptors are synthesized by autocrine and paracrine mechanisms. These ligands stimulate the STAT3-mediated upregulation of miR-92a and resulting downregulation of $p57^{Kip2}$ (p57).

Upper panel: MiR-92a is pivotal in maintaining podocyte tolerance to inflammatory insults. Among potential other actions, miR-92a eliciting repression of the CDK inhibitor $p57^{Kip2}$ unlocks cell cycle check points. This event, together with mitogenic stimuli elicited by these upstream pathways promotes podocyte proliferation resulting in proteinuria, destruction of the glomerular filtration barrier and declining renal function.

Lower panel: Anti-miR-92a strategy favors podocyte quiescence and tolerance to mediators of immune and mitogenic stress.

Figure 2f

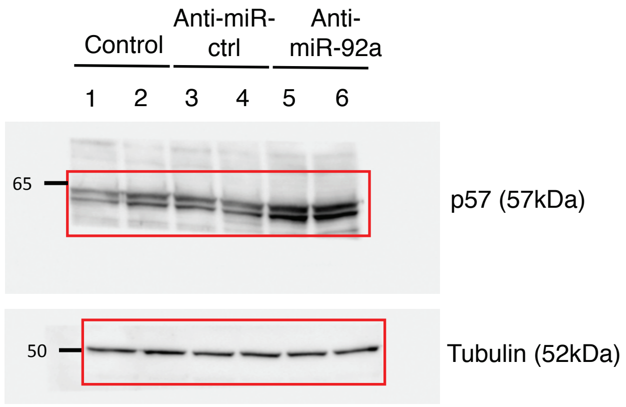

Figure 3a

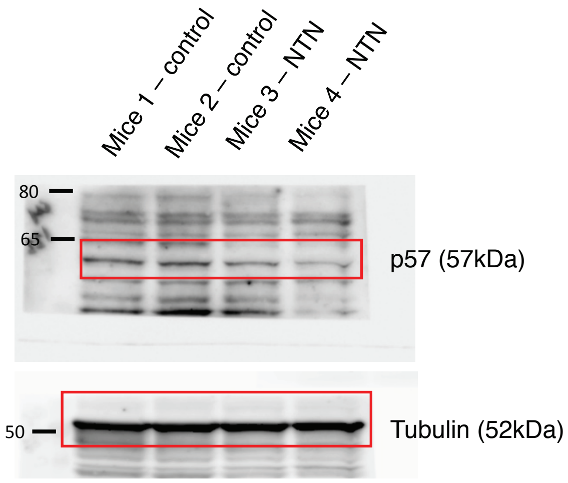

Figure S2a

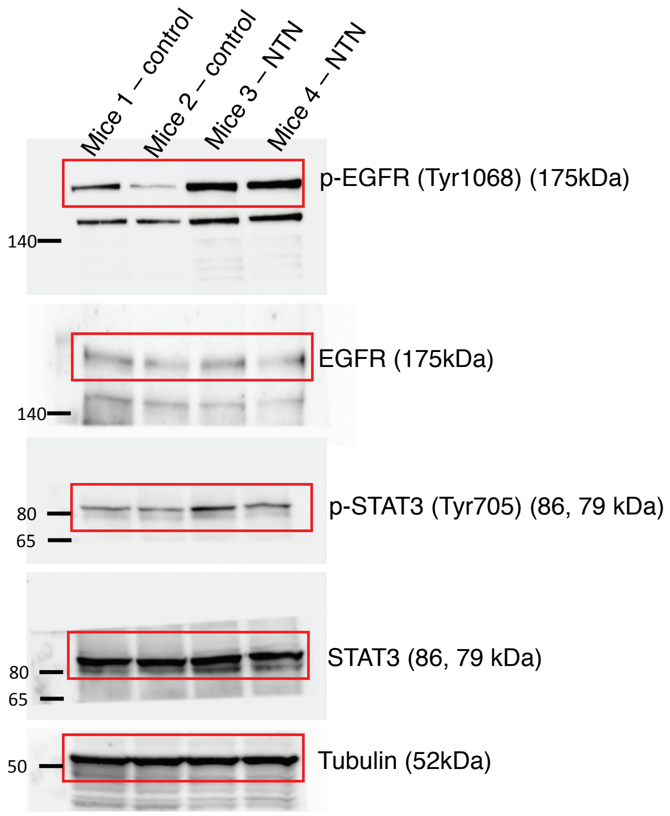

Supplementary Figure 10: ***uncropped scans of the most important western blots presented in previous figures accompanied by the locations of molecular weight markers.***
Red rectangles delineate displayed representative signals.

Figure S3b

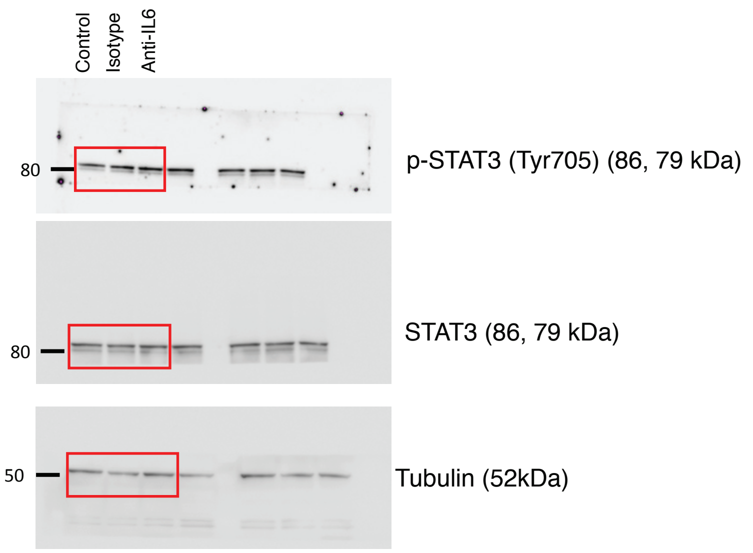

Figure S3d

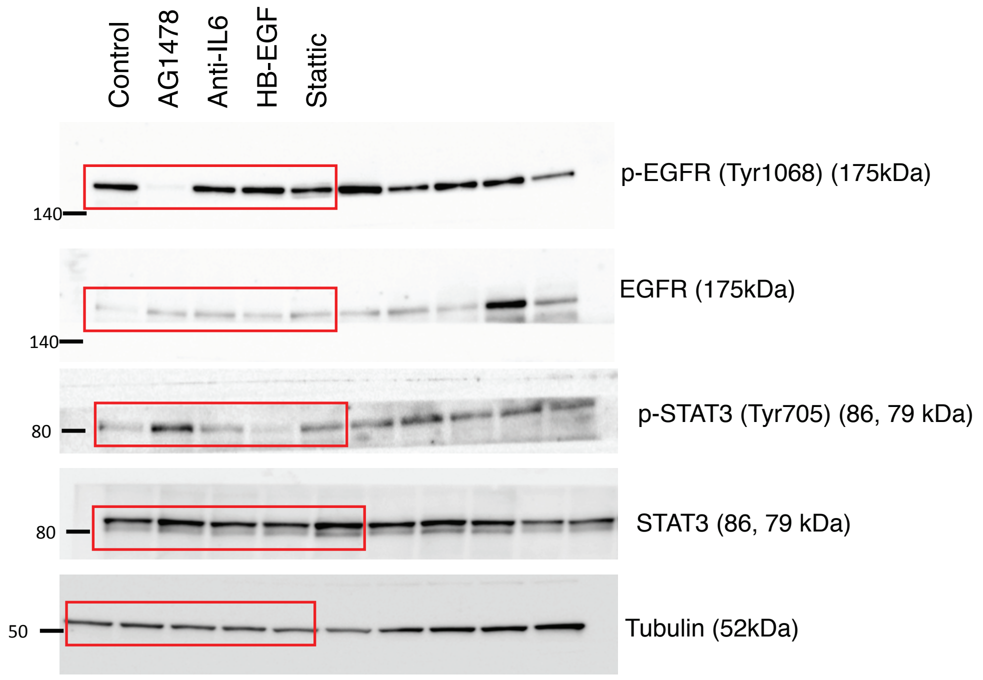

Supplementary Figure 10 continued: ***uncropped scans of the most important western blots presented accompanied by the locations of molecular weight/size markers***

Figure S4c

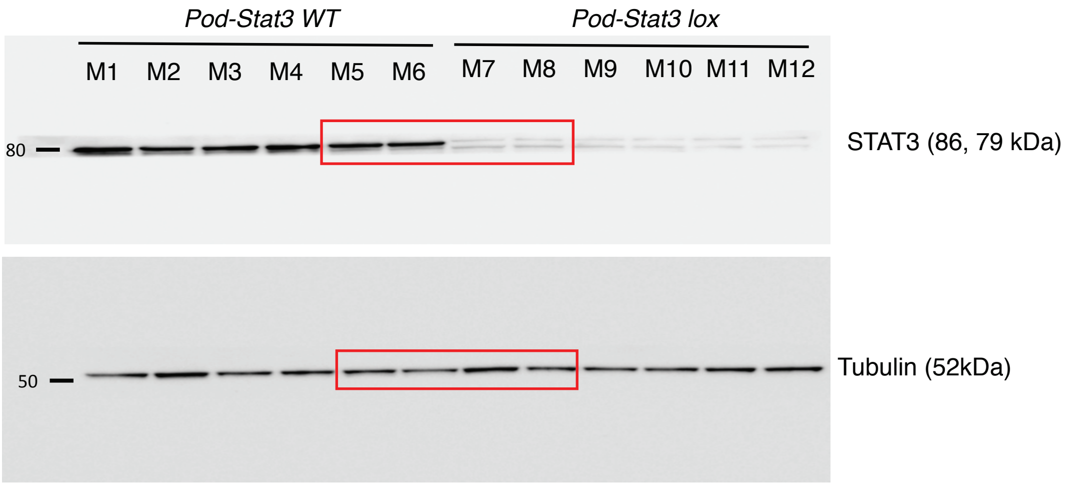

Figure S6d

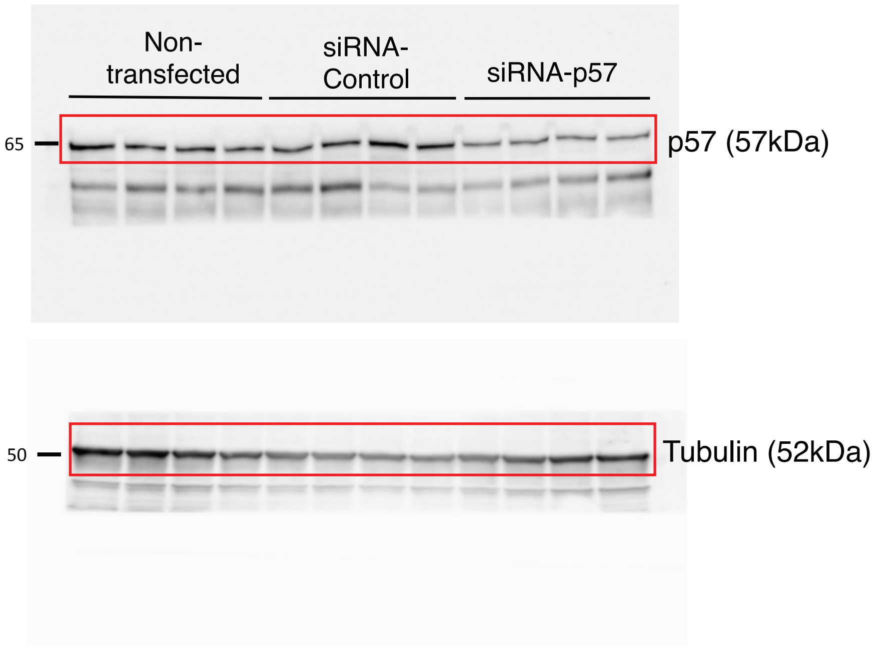

Supplementary Figure 10 continued: ***uncropped scans of the most important western blots presented accompanied by the locations of molecular weight/size markers***
